# A dataset of citizen science practitioners’ experiences and practices

**DOI:** 10.1016/j.dib.2024.110779

**Published:** 2024-07-31

**Authors:** Michael O'Grady, Eleni Mangina

**Affiliations:** School of Computer Science, University College Dublin, Belfield, Dublin 4, Ireland

**Keywords:** Citizen science, Participatory science, Open science, Data management

## Abstract

There has been renewed interest in Citizen Science (CS) in recent years as it offers an intriguing vision of enabling a scientifically literate population engage in scientific investigations and policy formation. Nonetheless, citizen scientists remain an understudied population, possibly due to the voluntary and part-time nature of their endeavours. Here, a dataset of CS practitioners’ experiences collected using an online survey is presented. The survey sample comprises 100 adults (18+) active in diverse CS projects. The survey contains 47 questions designed for quantitative analysis. Questions cluster around several broad themes - participant demographics, project profiles, experience in citizen science, data collection practices, management, dissemination, knowledge of open research principles, and training received. The dataset offers the potential for further empirical research or as a baseline for subsequent surveys, and will interest anybody planning a CS initiative. The questionnaire constitutes a ready-to-deploy instrument for additional country, region, or initiative-level surveys.

Specifications Table

**Table:** 

Subject	Environmental Science, Management, Monitoring, Policy and Law
Specific subject area	Citizen science
Type of data	Raw data in a table (Excel spreadsheet)
Data collection	Data was collected using an online survey implemented through Google Forms. Participants could only contribute once they confirmed they were over 18 years of age, understood the motivations behind the project, and gave permission to share the data. No identifiable data was requested. Data was stored at the authors’ institution only after participants pressed the submit widget on the final page of the survey.
Data source location	Institution: University College Dublin City/Town/Region: Dublin Country: Ireland
Data accessibility	Repository name: Zenodo Data identification number: 10.5281/zenodo.12548824 Direct URL to data: https://zenodo.org/doi/10.5281/zenodo.12548824
Related research article	M.J. O'Grady, E. Mangina, Citizen scientists - practices, observations, and experience, Humanit. Soc. Sci. Commun 11 (2024) 1-9. https://doi.org/10.1057/s41599-024-02966-x. [1]

## 1 Value of the Data

• Open datasets that report on studies of the participatory science community are rare.
• This dataset provides an in-depth snapshot of CS participants, their projects and activities, understanding of data practices, knowledge of open science, and training.
• Primary beneficiaries of this dataset are those who plan to launch and operate a participatory science initiative.
• Insights into how the crucial issue of data quality is perceived and practised may be gleaned.
• The dataset provides a baseline for longitudinal studies monitoring the practices of the CS community.

## 2 Background

Participatory science [2] involves the public contributing to scientific activities ranging from data collection to analysis to interpretation. Diverse models of participatory science exist, of which Citizen Science (CS) is one of the most common. Here, public members contribute to empirical science initiatives, often through data collection. More recently, the potential of the CS community to confront the perceived democratic deficit [3,4] has been considered [5].

CS participants have been studied through online questionnaires [6] and open-ended interviews [7]. However, little is known about their understanding of fundamental concepts such as open science, Responsible Research and Innovation (RRI) [8], and good data practices. In the latter instance, data is a cornerstone of CS and data quality is an omnipresent concern in conventional CS [9,10]. For communities seeking to influence policy definition [11], it is vital that they can provide data of adequate quality to form a credible, transparent, and robust evidence base. Typically, a professional scientist acts as a de facto quality controller. However, such expertise may be lacking for community-driven initiatives. Thus, to maximise the potential of CS, the training needs of CS participants, as well as their understanding, experiences, and perceptions, merit further investigation.

## 3 Data Description

This dataset is presented as a Microsoft Excel worksheet. The data is in raw format. Each column relates to one question, and that question's text forms the header. For clarity purposes, the questionnaire is also included in the supplementary material. Most questions are multiple-choice or checkboxes where more than one option can be selected. Some grid-style questions are also harnessed. All mandatory questions had to be completed to proceed from one form to another. One question was optional – about the country that the participant was from. Conditional branching occurred once in the questionnaire. When participants responded positively, they were asked to complete four more questions. All identifiable and personal data has been removed. With these exceptions, the dataset is complete.

This dataset contains data provided by 100 participants. Of these, 53% identified as female, 45% as male, and 2% preferred not to say. Table 1 illustrates the age profile, with the 35–44 profile being the largest sub-group. 95% of participants reported as being from Europe. Seventeen countries were represented, but 14% of participants did not declare their nationality. Ireland provided the most participants (36%), most of whom were involved in biodiversity recording.

**Table 1:** Participants’ age distribution.

Age	%
18–24	2%
25–34	13%
35–44	31%
45–54	22%
55–64	19%
65+	13%

88% of participants were involved in data collection, an activity synonymous with CS. In terms of roles, 50% defined themselves as an “Active Citizen Scientist”, while 40% categorised themselves as managers of a CS project. Overall, 63% contributed to decision-making in their projects.

A diverse spectrum of scientific domains was presented, with environmental science being the most popular (Table 2).

**Table 2:** Citizen Science domains where participants contribute**.**

Domain	%
Biodiversity	22%
Earth Science	24%
Environmental Science	33%
Life Sciences	4%
Social Science	8%
Space Science	3%
Other	6%

## 4 Experimental Design, Materials and Methods

The design of the survey questionnaire was influenced by project objectives and findings from related research in this domain [6,12]. The dataset was collected through an online survey. This survey was developed using Google Forms. It was subsequently translated into several languages - namely French, German, Greek, Italian, Polish, Portuguese, Spanish, and Turkish. Native speakers completed translations. However, the authors translated the responses as needed.

Adopting a convenience sampling approach, links to the survey were circulated widely using a combination of social media, online fora, and mailing lists. All channels were selected based on the expectation that their readership was predominantly CS participants. Potential survey participants were directed to the project website, where additional background and contextual information were available.

Before undertaking the survey, participants were requested to read some background material about the project's motivations. They were also informed that the dataset would be made openly available and that no personal or identifiable data would be requested. Participants could start the survey only when they agreed and confirmed that they consented to the conditions. As the survey was deemed low-risk, ethical exemption was granted. Participants were warned that completing the study would take at least 20 minutes. The survey was undertaken in Spring 2023, and 120 participants volunteered to contribute.

On analysing the data from a quality perspective, 20 contributions were found to lack consistency and exhibited internal contradictions. These were excluded, rendering the final dataset of 100 participants.

## Limitations

This survey is archetypal of online surveys. Thus, it encompasses many disadvantages synonymous with such surveys, including self-selection bias and an overall lack of control [13]. Measures were undertaken to ensure the integrity of the dataset, and the anonymity of participants may have contributed to more honest answers. Ultimately, it must be assumed that participants considered themselves citizen scientists, irrespective of their role in a particular project at the time of the survey. However, the risk of false or randomly selected answers is omnipresent.

The dataset is limited. One hundred participants is considered small for online surveys, reducing the potential for generalisation. While the decision to go deep rather than broad was a pragmatic design decision, the results must be interpreted more as indicative than general.

The participants are overwhelmingly from Europe. Thus, the dataset broadly reflects the experiences of those within a limited geographic area. The degree to which the findings may apply to the other regions of the global north, especially North America, where CS is well-established, is open to question. Moreover, a global south perspective is lacking, even though CS has a long tradition in South America.

From an inclusion perspective, participants were limited to those who were computer-literate.

## Ethics Statement

All research was performed according to relevant guidelines at University College Dublin, including, but not limited to, informed consent and data protection. The research was carried out in accordance with the Declaration of Helsinki as far as applicable to this type of study. As this survey was assessed as low risk, an exemption was obtained from the Office of Research Ethics at University College Dublin (LS-E-21-235-OGrady).

Informed consent—All participants provided informed consent. After reading the background, motivations, and data management policies, participants could either agree and proceed to the survey or disagree, in which case they could not. Participants further confirmed their agreement that the resultant dataset would be published as an academic publication.

## CRediT authorship contribution statement

**Michael O'Grady:** Conceptualization, Methodology, Formal analysis, Visualization, Writing – original draft. **Eleni Mangina:** Investigation, Formal analysis, Writing – review & editing.

## Data Availability

Survey of Citizen Scientists (Original data) (Zenodo).

## References

[1] O'Grady M.J., Mangina E. (2024). Citizen scientists - practices, observations, and experience. Humanit. Soc. Sci. Commun.

[2] Haklay M. (2018). Participatory citizen science. Citizen science: Innovation in open science. Soc. Policy.

[3] Mavrouli R., Van Waeyenberge A. (2023). EU Responses to the democratic deficit and the rule of law crisis: is it time for a (New) European exceptionalism?. Hague J. Rule Law.

[4] Liargovas P., Papageorgiou C. (2024). The European Integration.

[5] Roszczyńska-Kurasińska M. (2023). Beyond data: recognizing the democratic potential of 154 citizen science. IEEE Technol. Soc. Mag..

[6] Schade S., Tsinaraki C. (2016).

[7] Jones G.M., Childers G., Andre T., Corin E.N., Hite R. (2018). Citizen scientists and non-citizen scientist hobbyists: motivation, benefits, and influences. Int. J. Sci. Educ. B.

[8] Zwart H., Barbosa Mendes A., Blok V. (2024). Epistemic inclusion: a key challenge for global RRI. J. Responsible Innov..

[9] Jäckel D., Mortega K.G., Darwin S., Brockmeyer U., Sturm U., Lasseck M., Moczek N., Lehmann G.U., Voigt-Heucke H.L. (2023). Community engagement and data quality: best practices and lessons learned from a citizen science project on birdsong. J. Ornithol..

[10] Balázs B., Mooney P., Nováková E., Bastin L., Jokar Arsanjani J. (2021). The Science of Citizen Science.

[11] Guerrini C.J., Majumder M.A., Lewellyn M.J., McGuire A.L. (2018). Citizen science, public policy. Science.

[12] O'Grady M., Mangina E. (2022). Adoption of responsible research and innovation in citizen observatories. Sustainability.

[13] Andrade C. (2020). The limitations of online surveys. Indian J. Psychol. Med..

